# Healing After Dental Trauma: A Report of Two Cases on the Regeneration of Traumatized Immature Permanent Teeth

**DOI:** 10.1155/crid/2658507

**Published:** 2026-06-16

**Authors:** Neha Shrestha, Sumita Upadhyay, Nitesh Chaurasia, Parayash Dallakoti, Rasna Shrestha

**Affiliations:** ^1^ Department of Pedodontics and Preventive Dentistry, College of Medical Sciences, Kathmandu University, Bharatpur, Chitwan, Nepal, ku.edu.np; ^2^ Department of Pedodontics and Preventive Dentistry, Kathmandu University School of Medical Sciences, Kathmandu University, Dhulikhel, Nepal, ku.edu.np; ^3^ Department of Oral and Maxillofacial Surgery, Kathmandu University School of Medical Sciences, Kathmandu University, Dhulikhel, Nepal, ku.edu.np; ^4^ Department of Pedodontics and Preventive Dentistry, Nobel Medical College, Kathmandu University, Biratnagar, Nepal, ku.edu.np

**Keywords:** immature teeth, platelet-rich fibrin, regenerative endodontics

## Abstract

These case reports describe traumatic dental injuries affecting maxillary permanent central incisors in young children, which may lead to pulp necrosis with or without apical periodontitis. Immature teeth with necrotic pulp are very challenging to treat because of their short and thin dentinal walls, open apices, and broad root canals that make them weak and prone to fracture, which can ultimately render the tooth unrestorable and require extraction. Apexification has traditionally been the treatment of choice for such cases; however, recently, regenerative and revascularization procedures have shown encouraging results, including improved radicular dentin thickness and increased root length. The primary aim of this case report was to describe Regenerative endodontic procedures (REPs) performed on three post‐trauma immature permanent maxillary central incisors with necrotic pulp. Each tooth was treated using a different biocompatible material, all of which resulted in favorable clinical and radiographic outcomes. This report includes two patients aged 8–10 years, presenting with three maxillary central incisors diagnosed with pulp necrosis. All teeth were managed following the established REPs guidelines. Blood clot revascularization (BCR) was performed in two teeth, followed by placement of white, putty consistency mineral trioxide aggregate (MTA) in one tooth and capsule Biodentine in the other. In the third tooth, platelet‐rich fibrin (PRF) was used as the scaffold, followed by white putty consistency MTA placement. At the 12‐month follow‐up, both cases were clinically asymptomatic and radiographic evaluation showed advancing root development. The PRF treated tooth also exhibited a positive response to sensibility testing using an electric pulp tester. These case reports suggest that, with appropriate case selection, REPs can regenerate and revitalize post‐trauma immature permanent teeth with necrotic pulps and act as a beneficial alternative to apexification.

## 1. Introduction

Regenerative endodontic procedures (REPs) is defined as a biologically based approach aimed at restoring damaged tooth structures, including dentin, root, and the pulp dentin complex, in a physiological manner [1]. The primary objectives of REPs are the elimination of clinical signs and symptoms, resolution of periapical pathology, continued root maturation, and increased dentinal wall thickness to reduce risk of fracture in teeth with necrotic pulp and immature apices [1]. The conventional endodontic treatment in young permanent teeth is always challenging due to the absence of an apical constriction and the presence of thin, fragile radicular dentin, making it highly susceptible to breakage. Apexification has traditionally been employed in the management of immature permanent teeth; however, in recent years, REPs have demonstrated promising outcomes in the treatment of nonvital immature teeth [2].

The three essential ingredients of tissue regeneration are stem cells, scaffolds, and growth factors along with important effective removal of infection and necrotic tissue [3]. Even though REPs are now guided by standardized protocols, achieving consistent and predictable outcomes, is still a significant challenge. In addition, the selection of appropriate nonvital teeth is a primary hurdle, as the success of REPs depends on the presence of residual viable stem cells in the periapical tissues capable of supporting the tissue regeneration [4]. Although REPs are increasingly documented in the literature, their clinical predictability in cases of severe posttraumatic necrosis remains a subject of ongoing study. These two cases demonstrate a specific clinical lesson: the successful transition from a completely necrotic, traumatized state to functional root development in a high‐risk pediatric demographic. This report provides specific decision‐making insights into the comparative use of blood clots versus PRF as scaffolds, offering a protocol for achieving predictable biological healing in immature permanent teeth even when the initial clinical presentation is highly compromised.

## 2. Case Reports

### 2.1. Case 1

A 9‐year‐old boy presented with a history of trauma 3 months back, involving the maxillary right and left central incisors. Clinical examination revealed complicated crown fractures of both incisors, Teeth 11 and 21, as shown in Figure 1. Both teeth were tender to percussion and did not respond to pulp thermal tests (cold test and heat test) or electric pulp testing. Intraoral periapical radiographs (IOPAs) demonstrated immature roots in Teeth 11 and 21, consistent with Cvek Stage III root development [5], with approximately two‐thirds of root formation, as shown in Figure 2.

**Figure 1:**
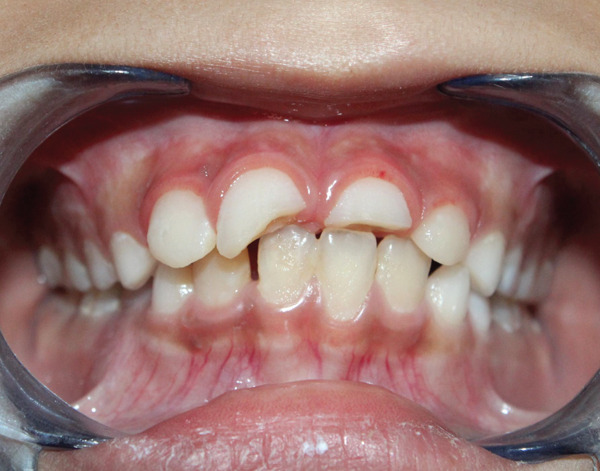
Complicated crown fracture with respect to 11 and 21.

**Figure 2:**
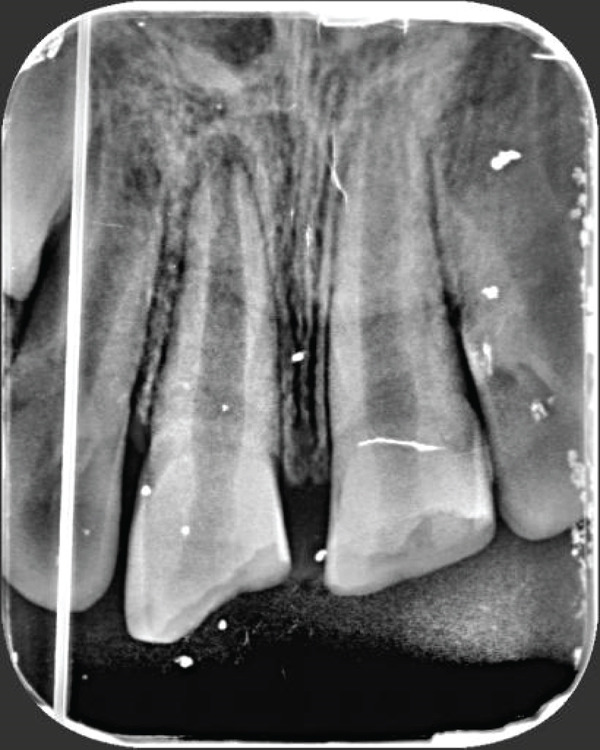
Intraoral radiograph of 11 and 21 showed wide root canal and an open apex.

All treatment options were explained to the patient′s parents, and BCR was selected as the treatment approach. Written informed consent for treatment and publication of clinical data was obtained from the patient′s parents, along with verbal assent from the patient. Participation was entirely voluntary and the guardians were informed of their right to decline publication without any impact on the patient′s care. The whole treatment was completed over three appointments.

All the procedures were performed following the guidelines and clinical considerations by American Association of Endodontists [6]. At the first appointment, local anesthesia was administered using 2% lidocaine with 1:100,000 epinephrine. Rubber dam isolation was achieved, and access cavities were prepared for Teeth 11 and 21, as shown in Figure 3. Working length was determined radiographically. The canals were irrigated with 20 mL of 1.5% sodium hypochlorite (NaOCl), followed by a final rinse of 20‐mL normal saline. To minimize the risk of apical extrusion, irrigation was delivered using either a closed‐end, side‐vented needle (positive pressure). The canals were then dried with paper points and triple antibiotic paste (TAP) consisting of metronidazole, ciprofloxacin, and cefaclor in a 1:1:1 ratio was placed as an intracanal medicament, and the access cavities were temporarily restored with 3M Cavit Temporary Filling Material [6].

**Figure 3:**
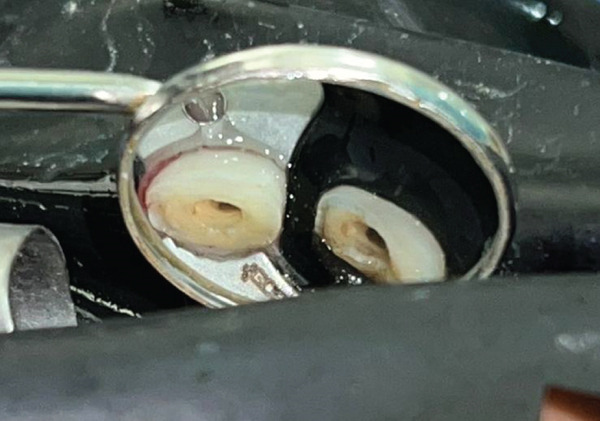
Rubber dam isolation and root canal access established with respect to 11 and 21.

After 4 weeks, right infraorbital nerve block using 2% plain lidocaine was administered for Tooth 11, and rubber dam isolation and reaccess were established. The canal was irrigated with 20 mL of 17% ethylenediaminetetraacetic (EDTA) acid with closed‐end, side‐vented needle and then dried with paper points. The working length was extended by 3 mm, and bleeding was induced in the apical region by over‐instrumentation. The canal was allowed to fill with blood up to the cemento‐enamel junction (CEJ), as shown in Figure 4. A 3–4‐mm layer of premixed white MTA putty was placed as a cervical barrier to provide a coronal seal and the access cavity was then temporarily restored with temporary filling material, Cavit (3M ESPE, St. Paul, Minnesota, United States) to prevent microleakage [6].

**Figure 4:**
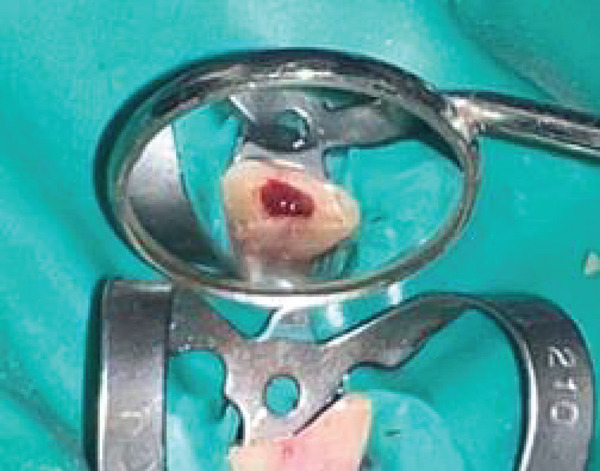
Blood clot revascularization—root canal filled with blood up to CEJ.

Three days later, a left infraorbital nerve block was administered for Tooth 21. The same clinical protocol including irrigation and medication used for Tooth 11 was also performed for Tooth 21. However, for 21, Biodentine (Septodont, Saint‐Maur‐des‐Fossés, France) was placed as the coronal sealing material over the blood clot. This was followed by temporary restoration using Cavit (3M ESPE, St. Paul, Minnesota, United States) as shown in Figure 5. MTA was employed in 11 for its excellent biocompatibility and proven long‐term success in achieving a seal. For 21, Biodentine was used as the bioactive sealing material. Although both materials offer similar regenerative outcomes, Biodentine was specifically chosen to mitigate the risk of cervical crown discoloration and induce faster setting of the material, which is a known limitation of traditional gray or white MTA. This choice was based on the clinical availability and requirement for superior esthetic outcomes in the anterior segment.

**Figure 5:**
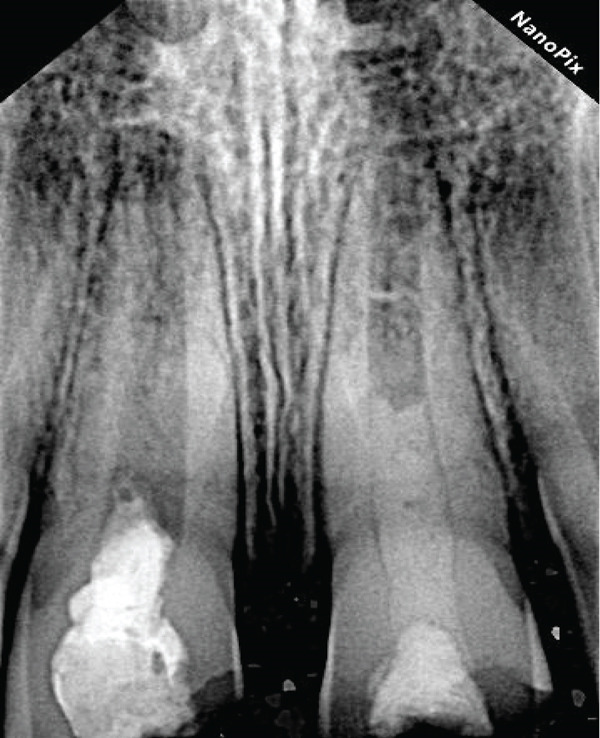
Intraoral periapical radiograph of 11 and 21 after blood clot revascularization, with MTA and Biodentine, respectively.

The case was followed routinely for up to 12 months. At the 12‐month follow‐up, both teeth were clinically asymptomatic and showed a positive response to the thermal sensibility test. A physiological response was elicited using a refrigerant spray for the cold test (Endo‐Frost, Roeko, Langenau, Germany) and a heated burnisher for the heat test. The radiographic evaluation was performed using standardized IOPAs with the parallel technique. Although quantitative volumetric analysis was not conducted, a qualitative comparative assessment of the follow‐up films revealed progressive apical maturation and increased dentinal wall thickness in both Teeth 11 and 21. These findings are consistent with the desired clinical outcomes of REPs [7], demonstrating continued root development and reduction of the apical diameter at the 12th‐month follow‐up as shown in Figure 6. These findings were expected to further improve during subsequent follow up visits.

**Figure 6:**
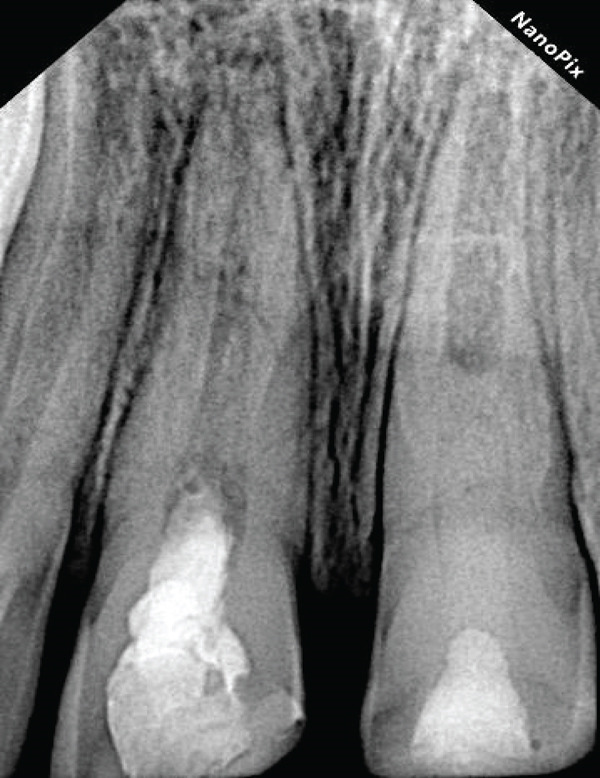
12‐month follow‐up, showing normal maturation of roots with thickening radicular dentinal walls, root lengthening, and decreasing apical diameter in 11 and 21.

### 2.2. Case 2

A 10‐year‐old boy presented to the Department of Pediatric and Preventive Dentistry with complaint of a broken tooth in the maxillary anterior region for the past 1 year. The patient had initiated primary endodontic treatment consisting of access opening at a local clinic 1 year back but was lost to follow‐up. However, he was motivated regarding esthetics concerns and thus visited our department. Clinical examination revealed a complicated crown fracture in Tooth 11 and an uncomplicated crown fracture in Tooth 21, as shown in Figure 7. Radiographic examination demonstrated arrested root development at Cvek Stage III [5], with a blunderbuss apex, as shown in Figure 8.

**Figure 7:**
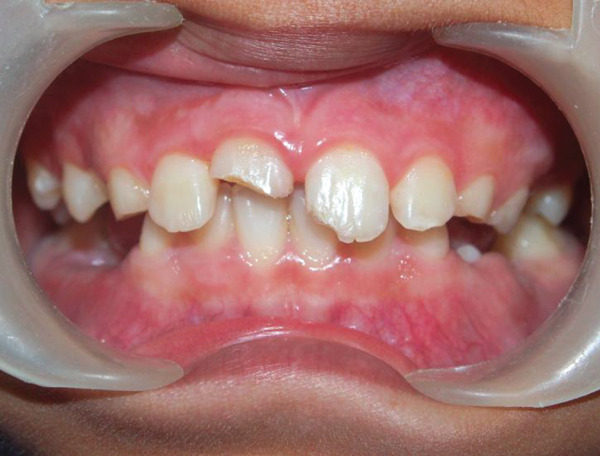
Complicated and uncomplicated crown fracture in 11 and 21, respectively.

**Figure 8:**
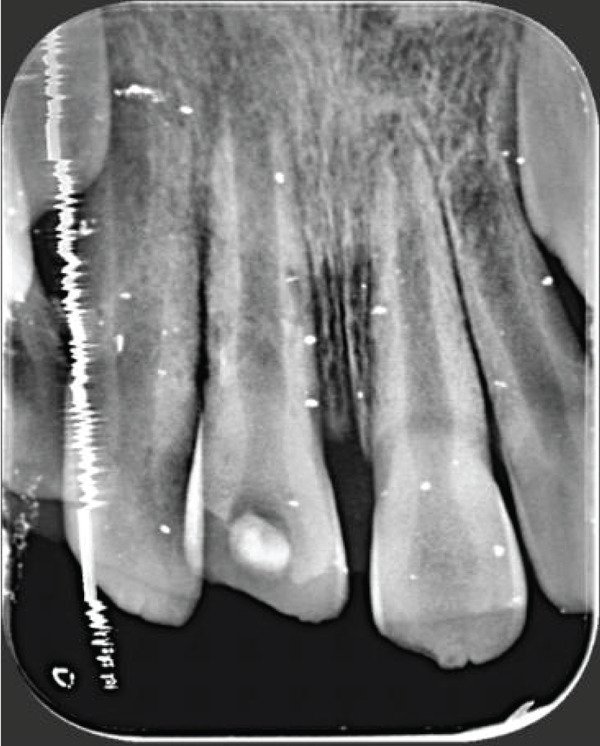
Intraoral periapical radiograph showed arrested development and wide root canal in 11.

All available treatment options and possible adverse effects were explained to the patient′s parents, and revascularization using the PRF protocol was selected to proceed with. Informed consent for the autologous PRF procedure and case publication was obtained from parents, alongside the patient′s assent. The consent involved a standard venipuncture for blood withdrawal, to which both the patient and guardians agreed voluntarily. The treatment followed the same standard protocol as in the first case until the second visit, where rather than inducing intracanal bleeding; the autologous PRF was utilized as the biological scaffold. Figures 9 and 10 illustrate first visit with working length determination.

**Figure 9:**
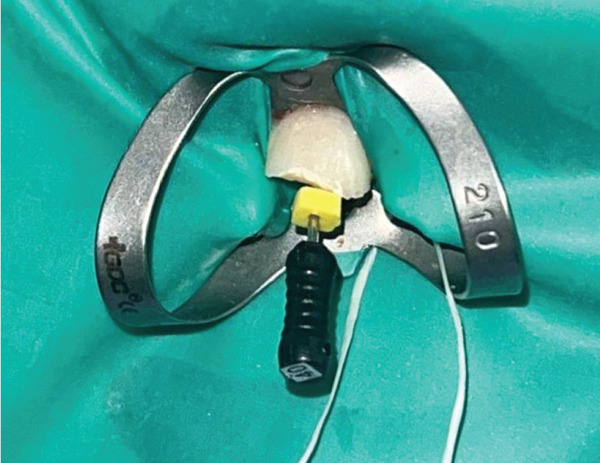
Working length determination for 11.

**Figure 10:**
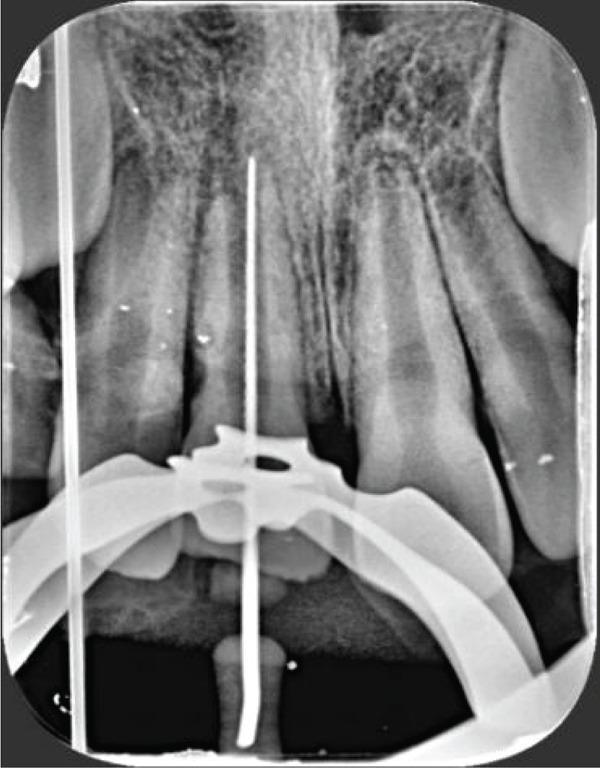
Intraoral periapical radiograph for working length confirmation.

At the second appointment, 10 mL of blood was withdrawn from the right median cubital vein, as shown in Figure 11, and centrifuged at 3000 rpm, as shown in Figure 12. Using sterile tweezers, the fibrin clot was retrieved, and an autologous fibrin membrane was extracted, as in Figures 13 and 14. Simultaneously, canal preparation was carried out in the same manner as described in the first case. The freshly prepared platelet‐rich fibrin membrane was then inserted into the canal and gently condensed below the level of the (CEJ) using finger pluggers, ensuring extension toward the apical region, as in Figure 15.

**Figure 11:**
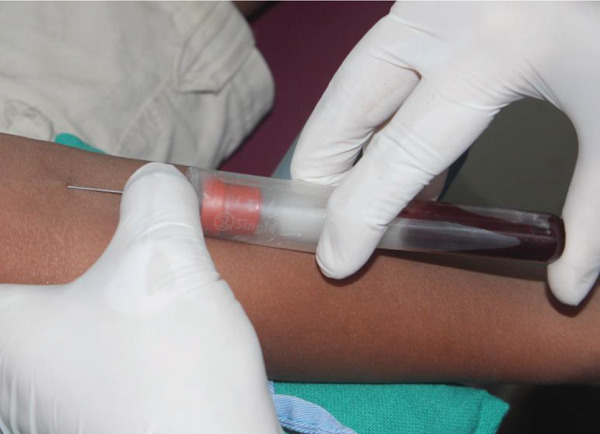
Right median cubital blood withdrawal for PRF preparation.

**Figure 12:**
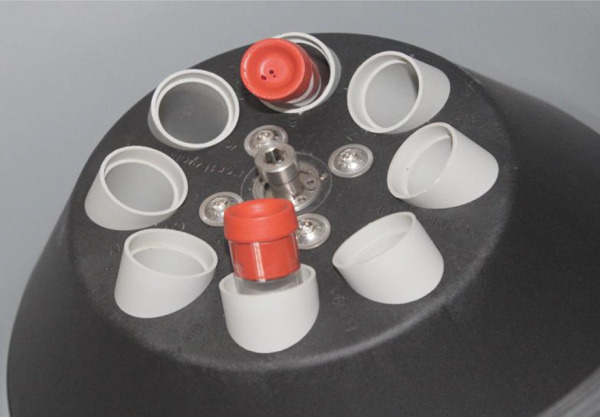
Blood centrifugation for PRF preparation.

**Figure 13:**
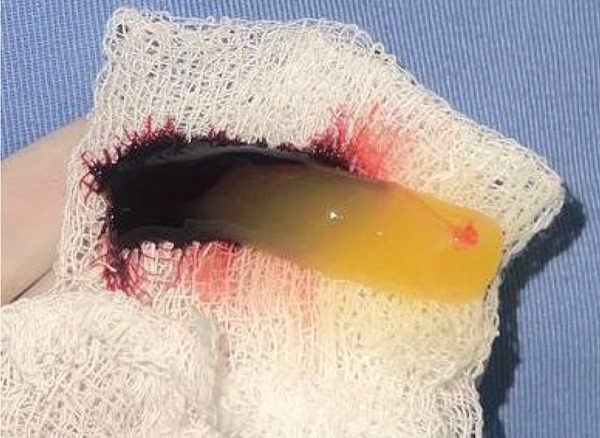
Removal of fibrin clot.

**Figure 14:**
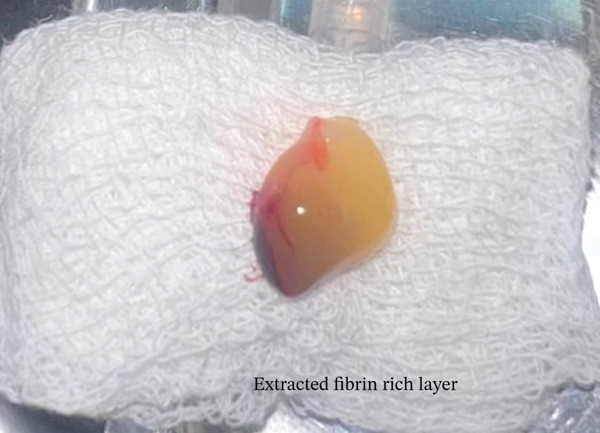
Extraction of platelet‐rich fibrin.

**Figure 15:**
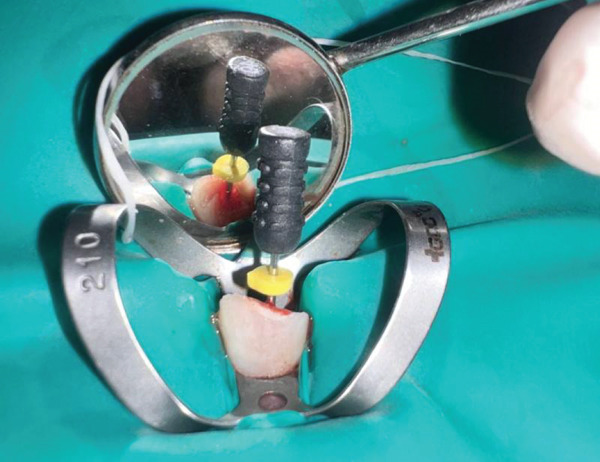
Placement of PRF into the canal up to CEJ with finger plugger.

Approximately 3 mm of premixed putty white MTA (CeraPutty, Meta Biomed, Cheongju, Korea) was placed over the PRF scaffold to achieve a coronal seal, followed by placement of a temporary restoration with Cavit (3M ESPE, St. Paul, Minnesota, United States) as shown in Figures 16 and 17. Three weeks later, for the final restoration of 11 and 21, composite buildup (Prime Bulk, Meta Biomed, Cheongju, Korea) was completed, as in Figure 18. At 12‐month follow‐up, the tooth was clinically asymptomatic and demonstrated a positive response to thermal sensibility test (cold and heat test). Radiographic evaluation also showed evidence of continued root maturation, shown by dome shaped apical closure with increased apposition of the radicular dentin, as shown in Figure 19. The tooth also elicited a positive response to electric pulp testing using an EPT device (Waldent, New Delhi, India) as shown in Figures 20, 21, and 22. The return of electrical sensibility in Tooth 11 confirmed the re‐establishment of functional neural pathways, achieving the tertiary goal of the regenerative protocol [7]. The comparison of the clinical features, their respective interventions and outcomes of both cases are summarized in Table 1.

**Figure 16:**
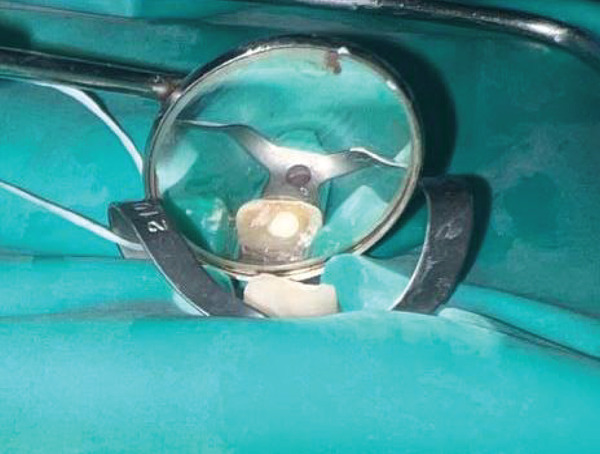
Canal sealed with MTA followed by temporary restoration.

**Figure 17:**
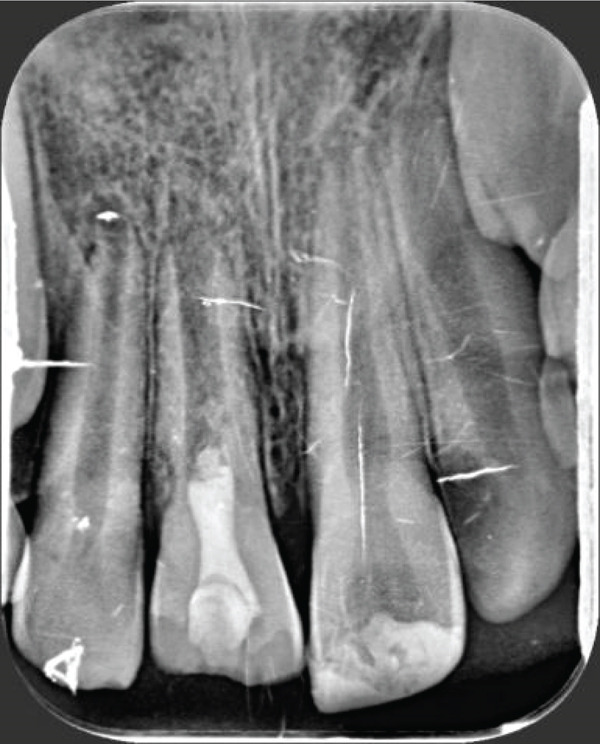
Intraoral periapical radiograph with coronal seal by MTA.

**Figure 18:**
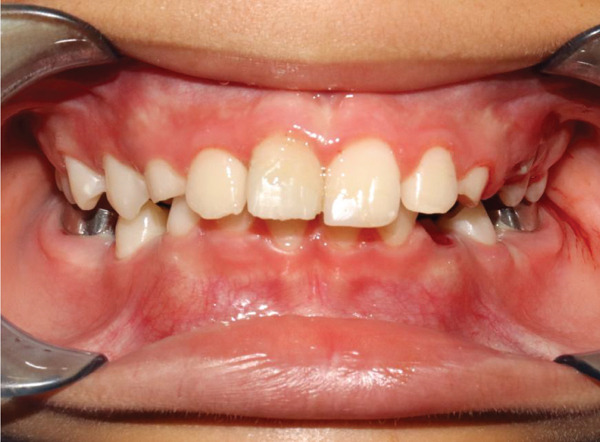
Composite build up restoration in 11 and 21.

**Figure 19:**
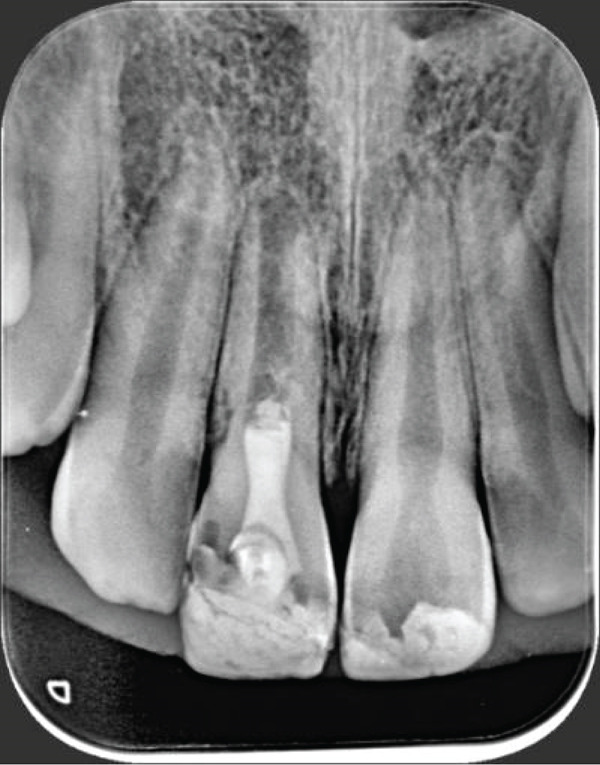
12‐month follow‐up with radicular dentinal walls thickening, root lengthening, and dome shape apical closure in 11.

**Figure 20:**
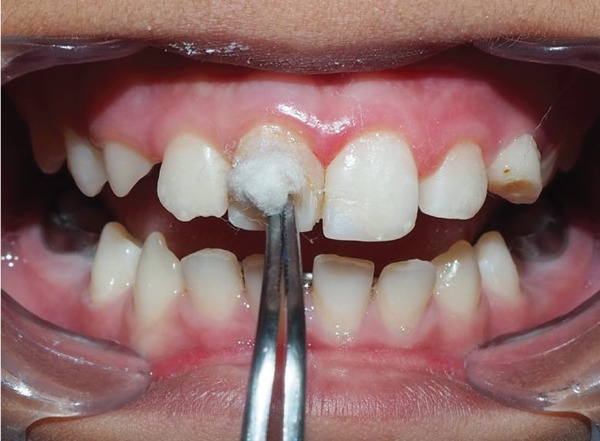
Positive response to thermal test (cold).

**Figure 21:**
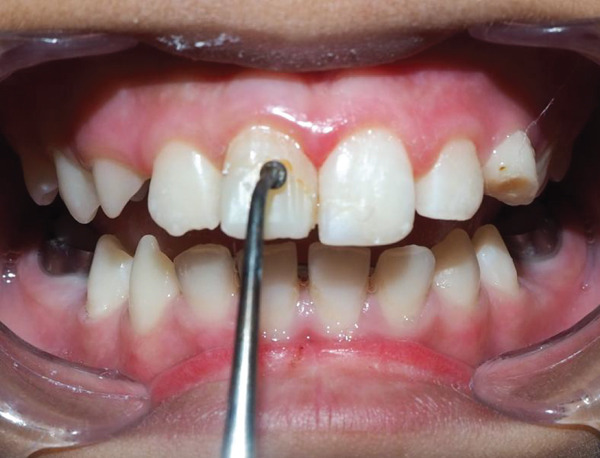
Positive response to thermal test (heat).

**Figure 22:**
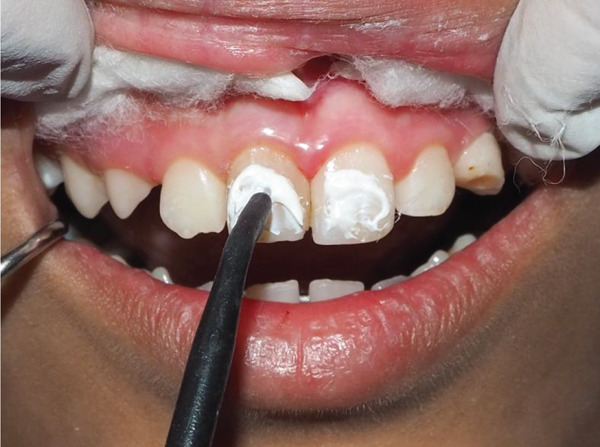
Positive response to electric pulp test.

**Table 1:** Summary of clinical features, interventions and 12 month outcomes.

Features	Case 1	Case 2
Age/gender	9/male	10/male
Etiology and tooth involved	Posttraumatic necrosis on 11 and 21	Posttraumatic necrosis on 11
Preoperative apex	Immature (open apex)	Immature (open apex)
Scaffold used	Induced intracanal blood clot	Platelet‐rich fibrin
Sensibility test (12 months)	Positive (cold/heat test)	Positive (cold/heat/EPT)
Bioactive material	MTA and Biodentine	MTA
Radiographic interpretation	Apical closure, root lengthening, dentinal thickness	Apical closure, root lengthening, dentinal thickness
Clinical status	Asymptomatic, no discoloration	Asymptomatic, no discoloration

## 3. Discussion

These case reports illustrate how REPs can preserve and revitalize traumatized immature teeth, potentially offering advantages over traditional apexification. Traumatic dental injuries account for approximately 92% of all oral injuries [8], and the reported prevalence of dental trauma in Nepal is 7.86% [9]. The most frequently affected teeth are the maxillary central incisors, which constitute nearly 80% of all dental injuries, followed by the maxillary lateral incisors [10]. Young children with immature permanent teeth represent the most vulnerable population. The management of young permanent teeth requiring endodontic intervention is particularly challenging, as trauma occurring during the early stages of root development can disrupt physiological apical closure [11]. Thin and fragile dentinal walls increase the risk of fracture during mechanical instrumentation and further complicate adequate canal disinfection [12]. Traditionally, apexification, apical barrier techniques, and REPs have been employed for the management of these teeth. Systematic reviews have reported success rates ranging from 87% to 93% for calcium hydroxide apexification and approximately 97.1% for MTA apexification [13]. A meta‐analysis comparing apexification and revascularization showed no statistically significant difference in overall treatment outcomes [14]. However, a recent 2025 meta‐analysis demonstrated that REPs resulted in a significantly greater increase in root length and root wall thickness, along with reduction in apical diameter, thus offering enhanced regenerative outcomes [15]. REPs were first introduced and later popularized by Banchs and Trope [16]. The mesenchymal stem cells involved in REPs may originate from the periapical tissues, dental pulp, or bone marrow. Stem cells from the apical papilla play a crucial role in regeneration following pulp necrosis. Successful regeneration depends heavily on effective canal disinfection prior to induction of bleeding or placement of a scaffold [17].

REPs are typically performed in two stages. The first stage includes local anesthesia, rubber dam isolation, and access cavity preparation with minimal or no mechanical instrumentation. This is followed by copious irrigation with 20 mL of 1.5% NaOCl and either 17% EDTA acid or normal saline with a closed end side vented needle in order to prevent the extrusion of the irrigating agents. The canals are then dried, and an intracanal medicament such as TAP or calcium hydroxide is placed before temporary restoration. After approximately 4 weeks, the second stage involves anesthesia and rubber dam isolation, followed by the passage of a pre curved K file beyond the apical foramen to induce bleeding. The canal is allowed to fill with blood to the level of the CEJ, creating a natural scaffold for regeneration [6]. Various scaffolds, including BCR, PRP, and PRF, have demonstrated promising in vitro and in vivo outcomes [18]. In Case 1, conventional blood clot was utilized as the scaffold, representing the gold standard for initiating the regeneration through the release of endogenous growth factors. In Case 2, PRF was selected as an autologous scaffold to enhance the regenerative potential. The choice of PRF in the second case was clinically supported by the patient′s high level of cooperation for venipuncture and parent′s positivity toward the new protocol.

Platelet concentrates provide a high concentration of growth factors that enhance angiogenesis and tissue vascularization giving rise to PRP, the first generation platelet concentrate [19], but was associated with the risk of antibody formation and coagulopathies due to the use of bovine thrombin. Consequently, PRF was developed as a second generation concentrate that is completely autologous, does not require anticoagulants, and provides sustained release of growth factors, resulting in improved clinical outcomes [20].

Several studies have reported accelerated root development and regeneration with platelet concentrates compared with blood clot alone. A meta‐analysis by Murray [21] reported apical closure success rates of 85.1% for platelet‐rich plasma, 85.2% for platelet‐rich fibrin, and 58.8% for blood clot revascularization. For root lengthening, success rates were 64.1% for blood clot revascularization, 64.2% for platelet‐rich plasma, and 74.1% for PRF platelet. These findings suggest that although BCR remains the most commonly used technique, PRF and PRP may result in more predictable apical closure [21]. Despite these advantages, the use of platelet concentrates remains limited due to the need for blood collection, increased procedural complexity, additional time, and higher cost [22].

Intracanal medicaments commonly used in REPs include calcium hydroxide and TAP. Calcium hydroxide is favored for its antimicrobial properties and its ability to promote attachment of human apical cells to root dentin [23]. However, concerns regarding soft and hard tissue calcification have led some clinicians to prefer TAP for more effective disinfection [6]. The American Association of Endodontists has outlined three goals for successful regenerative treatment. The primary goal is elimination of clinical symptoms and evidence of periapical healing. The secondary goal is continued root development, including increased root length and dentinal wall thickness. The tertiary goal is a positive response to sensibility and vitality testing [7]. In the present case report, the teeth treated with BCR achieved the secondary treatment goals, whereas the tooth treated with PRF achieved the tertiary goal [7]. These findings support existing evidence that REPs can provide clinical outcomes that are beneficial to patients when compared with conventional apexification. The radiographic progression observed in both cases—specifically the increase in dentinal wall thickness and the subsequent apical closure—serves as a primary indicator of successful biological healing. In Case 1, the use of a traditional blood clot scaffold provided a sufficient fibrin matrix for migration of mesenchymal stem cells from the apical papilla, leading to satisfactory radicular development. In Case 2, the placement of PRF predictably provided the regenerative timeline as a result of the sustained release of autologous growth factors from the PRF matrix that likely provided a more stable environment for cell proliferation compared with the spontaneous blood clot in Case 1. Furthermore, the transition from negative to positive sensibility in both cases suggests a successful reinnervation of newly formed intracanal tissue. It is noteworthy that although radiographic evidence of root lengthening was the primary goal, the clinical stability and lack of postoperative symptoms over 12 months confirm that the choice of coronal seals like MTA and Biodentine effectively prevented microleakage suggesting that the biological success of regeneration is as much dependent on the quality of coronal hermetic seal as it is on the intracanal scaffold. A significant limitation of this case report is the reliance on pulp sensibility testing, specifically cold and electric pulp tests, to evaluate clinical success. Although both cases exhibited a return of sensory response at 12‐month follow‐up, these results are not definitive evidence of histological pulp vitality or complete tissue regeneration. Sensibility tests do not confirm the presence of an established microvascular supply or the exact nature of newly formed intracanal tissue. However, in a clinical setting, where histological verification is not feasible, these positive outcomes are regarded as markers of neurosensory recovery and functional success rather than absolute proof of pulp vitality. With further controlled clinical research and standardized protocols, revascularization techniques may eventually replace traditional treatment approaches for immature necrotic permanent teeth.

## 4. Conclusion

These cases illustrate that regenerative endodontics is a viable alternative to traditional apexification for immature permanent teeth with posttraumatic necrosis. The clinical contribution of this report lies in the demonstration of continued root lengthening and dentinal wall thickening showing true biological healing achieved through appropriate case selection. By shifting the focus from simple canal obturation to the recruitment of apical stem cells, clinicians can ensure long‐term tooth survival and structural integrity in traumatized young permanent teeth. This highlights the necessity of adopting regenerative protocols as a primary treatment standard for necrotic immature teeth.

## Author Contributions

Neha Shrestha: examination of the patient, investigation, treatment planning, management of the patient, follow‐up, literature review, and writing—original draft; Sumita Upadhyay: treatment planning, investigation, and supervision; Nitesh Chaurasia: treatment planning, PRF preparation, and supervision; Parayash Dallakoti: supervision and writing—review and editing; Rasna Shrestha: supervision and writing—review and editing.

## Funding

No funding was received for this manuscript.

## Consent

Written informed consent was obtained from the legal guardians (parents) of both patients for the publication of these clinical cases, including all accompanying clinical photographs and radiographic images. A copy of the written consent is available for review by the editor in chief of this journal.

## Conflicts of Interest

The authors declare no conflicts of interest.

## Data Availability

The data supporting the findings of this case report are not publicly available due to patient privacy and confidentiality. However, clinical information can be made available from the corresponding author upon reasonable request and with appropriate permission.
